# Ritual uses of palms in traditional medicine in sub-Saharan Africa: a review

**DOI:** 10.1186/1746-4269-10-60

**Published:** 2014-07-23

**Authors:** Marta Gruca, Tinde R van Andel, Henrik Balslev

**Affiliations:** 1 Department of Bioscience – Research Group Ecoinformatics and Biodiversity, Aarhus University, Build. 1540, Ny Munkegade 114, DK-8000 Aarhus C, Denmark; 2 Naturalis Biodiversity Center, Leiden University, P.O. Box 9517 2300 RA Leiden, the Netherlands

**Keywords:** Arecaceae, Magic plants, Treatment, Healing, Sacred places, Witchcraft

## Abstract

Palms (Arecaceae) are prominent elements in African traditional medicines. It is, however, a challenge to find detailed information on the ritual use of palms, which are an inextricable part of African medicinal and spiritual systems. This work reviews ritual uses of palms within African ethnomedicine. We studied over 200 publications on uses of African palms and found information about ritual uses in 26 of them. At least 12 palm species in sub-Saharan Africa are involved in various ritual practices: *Borassus aethiopum*, *Cocos nucifera*, *Dypsis canaliculata*, *D. fibrosa, D. pinnatifrons, Elaeis guineensis, Hyphaene coriacea, H. petersiana, Phoenix reclinata, Raphia farinifera, R. hookeri,* and *R. vinifera.* In some rituals, palms play a central role as sacred objects, for example the seeds accompany oracles and palm leaves are used in offerings. In other cases, palms are added as a support to other powerful ingredients, for example palm oil used as a medium to blend and make coherent the healing mixture. A better understanding of the cultural context of medicinal use of palms is needed in order to obtain a more accurate and complete insight into palm-based traditional medicines.

## Background

Traditional medicines in rural sub-Saharan communities recognize that the occurrence of disease can result from the intrusion of negative supernatural forces [1,2]. These forces are often defined as witches, sorcerers, broken taboos, displeased ancestor spirits or deities [3-8]. Afflictions which are mostly related to the action of the malevolent forces are either serious and chronic or emerging suddenly and unexpectedly [4-7]. The patient is often considered a victim, and the therapy must heal not only physical symptoms but also social relationships to liberate the patient from suffering [9]. Thus, traditional healers often apply divination and various rituals in order to understand the overall significance of a healing process and counteract its cause. As a consequence, traditional remedies are not merely used for curing a disease, but are also used to obtain protection or to overcome curses [3,7,10,11].

Palms (family Arecaceae) are prominent in traditional cultures as a source of raw materials for consumption, construction, and other functions of daily life [12-16]. Traditional remedies are derived from palms throughout the tropics and subtropics to cure many disorders [17-21]. Since palms are part of the everyday life of nearly all rural people in Africa, it may be expected that they are also important in the spiritual framework of rural life in Africa. Even though many studies report ethnomedicinal uses of African palms, from the late nineteenth century [22,23] to very recent times [24]– especially recent studies pay little attention to rituals. In the latest studies on African traditional medicine palms are included among raw lists of plants used for specific ailments [20,25-31]. Detailed preparation and application of palm remedies are rarely mentioned [32-36]. While these types of studies may be useful when searching for potential modern drugs, they do not reveal the ideas underlying the use of the cited medicines nor do they explain why certain plants were selected for a ritual, or their exact therapeutic practice. On the other hand, most of the recent ritual palm use records came from anthropological studies, where the emphasis was put on the explanation of the ritual itself, but the botanical species was not defined [37-39].

Here we focus on palm-derived African ethnomedicine that includes ritual elements. By ritual (or magical treatment) we understand any medicinal practice involving objects (e.g. palm nuts) or behaviors (e.g. incantations) believed to have some healing powers and/or ability to counteract or influence the actions of malevolent forces.

We argue that we can only have an accurate insight into traditional medicine if we understand the cultural context of medicinal use of palms (and other plants). In this perspective we address the following specific questions: Which palm species take part in rituals or specific ceremonies? Which palm parts are mostly used? And finally, are palms present in the spiritual framework of African traditional medicines today?

## Methods

In total 26 scientific papers and books on African traditional medicine provided information on ritual uses of palms in Sub-Saharan Africa. This information was extracted and listed with scientific names, plant parts used and detailed use description (Table 1). Our bibliographic search employed several databases, including PubMed, Embase, and Google Scholar. In addition, we conducted a dedicated search with search engines of the State and University Libraries of Aarhus, National Library of Denmark and Copenhagen University Libraries, Mertz Library, and Harvard University Libraries where most of old, limited access literature was studied.

**Table 1:** Ritual uses of palms in traditional medicine in sub-Saharan Africa, including scientific plant names, plant parts used and detailed use description

**PALM SPECIES**	**PART USED (MENTIONED)**	**MEDICINE USED FOR/ACTIVITY**	**PREPARATION**	**APPLICATION**	**COUNTRY**	**ETHNIC GROUP**	**REFERENCES**	**NOTES**
*Borassus aethiopum*	root	Epilepsy	maceration	body bath	Togo		[65]	the author adds that epilepsy is believed to occur mostly during the full moon (10th to 15th days of a month)
*B. aethiopum*	seed	Scarification	seeds hollowed out	used as containers for a charred medicinal mixture called 'katala' in Haussa. This mixture was rubbed into skin incisions during scarification practices	Ghana	Ghana Haussa	[11]	
*B. aethiopum*	root	Any disease caused by a curse	decoction	drunk	Ghana	Kokomba	[Gruca,unpublished]	
*Cocos nucifera*	fruit	skin rash due to HIV/AIDS			Kenya	Suba, Luo	[46]	the disease is locally known as ‘chira’, and its etiology is related to the transgression of principles governing sexuality or seniority; for example: adultery committed during a wife’s pregnancy, having sexual intercourse during the harvest, or failure to observe the proper separation of sexuality between generations
*C. nucifera*	whole plant (palm tree)		planted at sacred places		Madagascar	Betsimisaraka	[13]	
C. nucifera* + Elaeis guineensis*	seed (coconut) + fruit (palm oil)	miscarriage/preventive	one tortoise is roasted with water inside a coco-nut along with half a bottle of **palm oil**. All is roasted until it is almost burnt and then ground to powder	the powder is used in a corn flour pudding, which a woman should take on rising and going to bed throughout the course of one menstruation. The man should sleep with her five days after she has finished menstruating	Nigeria	Yoruba	[47]	for Yoruba tortoise is a symbol of a prostitute
C. nucifera* + Unidentified palm	seed shell + sap (palm wine)	offering		coconut shells filled with palm wine placed on ancestors’ graves as an offering	Kenya	Mijikenda	[62]	
*E. guineensis*	root	epilepsy	powder, decoction or burnt powder	orally	Togo		[65]	the author adds that epilepsy is believed to occur mostly during the full moon (10th to 15th days of a month)
*E. guineensis*	seed (nut)	mental fatigue	a fresh **nut** easy to pound or smash is mixed together with leaves from *Hibiscus surattensis* L.*, Asystasia gangetica* T.Anderson*, Musa x sapientum* L., NGONGOA, Lopèto and *Cyperus articulates* L.	the mixture is rubbed on the body of the patient. The patient should be facing the sun during the treatment and pronounce “wishes” of good luck. The residues are put under the patient’s pillow. If he dreams of a young girl with erected breasts, there is hope for cure. He should not wash himself during the rest of the treatment day	Cameroon		[51]	
*E. guineensis*	*guineensis* seed (nut)	oracle rituals	**palm nuts** are used for ritual usages going as far as to be made sacred at the oracle *Fa* (Fon), *Ifa* (Yoruba), *Afan* (Ewe) consulted very often when looking for the causes of illnesses and of fate dreams		Benin	Fon	[51]	The author mentions Togo and Benin - ethnic groups were assigned to these countries
						Yoruba		
					Togo	Ewe		
*E. guineensis*	leaf (twig)	vulnerability	ill people carry pieces of **palm twigs** around the neck or arm to get invulnerability		Togo, Benin		[51]	
*E. guineensis*	root	keeping away bad spirits	**roots** are associated with the resin from *Daniellia oliveri* (Rolfe) Hutch. & Dalziel and *Commiphora Africana* (A. Rich.) Endl to keep away the bad spirits		West Africa		[51]	
*E. guineensis*	infructescence	make children walk	empty **infructescences** of *Elaeis guineensis* alone or mixed with ginger (*Zingiber officinale* Roscoe) are burned and applied as magical medicine in the form of an enema to small children to encourage them to walk at an early age		Ghana		[van Andel, unpublished]	
*E. guineensis*	inflorescence	drive away bad spirits	**inflorescences** from *Elaeis guineensis* are burned so the smoke drives away bad spirits		Ghana	Akan	[7,11]	
*Elaeis guineensis* var. idolatrica	whole plant (palm tree)	sacred place	the **palm** is protected as sacred where ever it grows because it is seen as the realization on earth of the god *Fa*. Nobody is allowed to cut it down or to use its fruits for making oil. The ritual use of these palms is reserved for soothsayers called *bokonon*		Benin		[71]	
E. guineensis*	fruit (palm oil)	backache	the doctor goes in the early morning to where the mortar stands. He gets a women to shift its position, digs down and removes a piece of any root he finds there, then he scraps some dirt from the base of the mortar itself. The scrapings from the root and the earth are mixed with **palm oil** in a potsherd	the doctor makes the patient lie across the hole where the root was removed, then he makes three lines of cuts with the razor across the patient's back where he felt pain and rubs medicine into the incisions. After that the patient has to pull himself upright by means of the pounding pole that usually goes with the mortar. He must then walk to his hut, and leave the pounding pole upright against a tree near his hut and never let it lie on its side	Zambia	Lunda	[9]	author: “the treatment for backache appears to be almost entirely magical”
E. guineensis*	fruit (red palm oil)	backache	first the doctor prepares the following: a part of a broken hoe blade and a portion of the splintered wood from a tree that has been struck by lightning. After that he goes to an old village site to the place where a mortar once stood for pounding cassava roots and grain. There he digs and removes the first root he finds (any species of tree). The patient is brought to lie under the *Diplorhynchus condylocarpon* (Müll.Arg.) Pichon tree that is naturally bent. The doctor scrapes off bark from underneath the bend in the tree, and also collects some scrapings from the upper side. Then he places a pounding pole at right angles to the tree. Then he adds to the medicine some scrapings from the top of a tortoise’s shell. Then the doctor brings a potsherd in which he puts **red palm oil**. He scrapes some iron dust from the broken hoe on to the oil. Then he burns the piece of lightning-struck tree and adds its ash to the mixture. In go the tortoise shell scrapings and finally the scrapings from the tree. The medicine is thoroughly mixed with the oil	the doctor makes two or three lines of small incisions across the patient’s back, where the pain is. When the blood begins to ooze, the doctor rubs the medicine into those cuts. Afterwards the doctor takes the pounding pole and presses it on the patient’s back where the incisions are. He presses the pestle lengthwise on the back with both hands. Subsequently the patient has to hold the pounding pole up vertically and go under the crook in the *Diplorrhyncus condylocarpon* tree. The patient has to straighten himself up, with the help of the pounding pole and push the tree up with his back. Then he must address the tree: “I have already left this disease with you. I must go home feeling no more pain, because I have left it already with you”. The patient returns home bearing the pounding pole	Zambia	Lunda	[9]	symbolic explanation of the ritual from the author: broken hoe blade snaps suddenly when people are digging; in the same way the person with backache feels as though he has suddenly been broken. A meal mortar is used because of the pounding, this represents hitting and having backache is like being hit very hard. A tortoise shell is used because it is hard and this medicine strengthens the back. The tree used has a white gum so it is white or lucky tree, the whiteness of the tree gives the patient health (color symbols among Ndmebu)
E. guineensis*	fruit (palm oil)	medico-magic	leaves of *Hyptis lanceolata* Poir. mixed with **palm oil**		Gabon	Masango	[49]	
E. guineensis*	seed (palm kernel oil)	scarification wounds	two loops of the split vine used, one left with a flat sharp edge for scrapping off the pus, the other pounded to make a soft brush loop with which the dressing is completed. The juice of *Rothmannia whitfieldii* (Lindl.)Dandy (cited as *Randia malleifera*) is rubbed in to cause a slight formation of keloid	The boy is told to lie on his mat first on one side, then on his face, then on the other side, then on his back, changing his position often to avoid uneven scarring, and bad sores. After a few days the wounds are dressed with **palm kernel o**il applied with a brush of owl's feathers	Liberia	Mano/Poro	[48]	
E. guineensis*	fruit (red palm oil)	fractures	a stick from each of the following trees or shrubs is calcined: cited as *Ricinodendron africanum* Pierre ex Pax, *Dracena* sp., *Whitfieldia lateritia* Hook. and any small twig broken over with the break healed so that the stick is growing in the twisted or bent position. The calcined wood is powdered and mixed with the **red palm oil**	the ointment is rubbed on the area over the fracture	Liberia	Mano	[48]	
E. guineensis*	fruit (red palm oil)	hiccough	a whole vine of *Clerodendron* sp. is calcined and beaten to powder. This powder is kept in a small horn, and a small amount mixed with **red palm oil**	eaten	Liberia	Mano	[48]	author: “charred drug is magical”
E. guineensis*	fruit (red palm oil)	palpitation	an inflorescence of *Costus* sp*.* is peeled; then a handful of *Harungana madagascariensis* Poir. buds is added - all beaten up in a mortar. Some of the mixture is put in an iron spoon with **red palm oil**, four pebbles are heated in the fire (three if for a woman) and dropped into the spoon	the patient licks the spoon	Liberia	Mano	[48]	numeric symbols
E. guineensis*	fruit (palm oil)	heart trouble, rapid pulse	a young shoot of unidentified plant is beaten up to a pulp and put in the spoon with a little **palm oil**. Three (or four) pebbles are heated in the fire and added to the spoon, stirred until cool - all done in the morning before the patient has eaten	the patient takes the contents of the spoon into his mouth spitting out the stones far away and swallowing the pulp. What is left on the spoon is rubbed over the pericordium	Liberia	Mano	[48]	numeric symbols
E. guineensis*	fruit (red palm oil)	rheumatism (due to yaws)	a small horn is filled with powdered charcoal from various plants mixed with **red palm oil** and leaves beaten to pulp		Liberia	Mano	[48]	author's explanation of symbolism: 'This is an example of blending "male" and "female" elements in a mixture to form a more powerful medicine. The leaves and bark of living plants and the red palm oil are supposed to represent the active male elements; the charred stems of other plants and "burned" oil … are supposed to represent the attenuated, magical, kore zxpreventive female elements’
E. guineensis*	fruit (palm oil)	respiratory pain due to pleurisy	a doctor takes a handful of *Bidens pilosa* L. (cited as Spanish needle), burns to ashes and mixes the ashes with **palm oil**	doctor and the patient sit facing each other, a doctor rubs the ointment on his hands, make two false passes around the patient's chest from back to front, then with the third he rubs hard (or fourth if it is a man). He gets a good hold for this last rub to lift a tremendous weight, pulls forward, and with what seems to be a great effort, rubs the sickness out and wipes it off on a bit of trash which he throws away	Liberia	Mano	[48]	numeric symbols
E. guineensis*	fruit (red palm oil)	influenza	a handful of thorns of *Combretum grandiflorum* G.Don is burned to charcoal in a pot, then heated with **red palm oil**	used to anoint the ankles, knees, and elbows	Liberia	Mano	[48]	"this remedy was originally revealed in a dream, probably suggested by the flaming suddenness of blooming of the great red panicles of this vine as resembling the appearance of the epidemic."
E. guineensis*	fruit (palm oil)	acute hepatitis	a piece of a large shelf fungus shaped like a liver is charred, powdered and mixed with **palm oil**	rubbed over the liver	Liberia	Mano	[48]	shape of the fungus is the shape of the organ cured
E. guineensis*	fruit (red palm oil)	coma	a knot of the parasite *Loranthus micranthus* Hook.f. where it joins the host branch is calcined and triturated in an iron pot. The black powder is mixed with **red palm oil**	rubbed on the patient's cheeks toward the mouth and he will talk	Liberia	Mano	[48]	
E. guineensis*	fruit (red palm oil)	coma	a medicine to give the cow's tail (see application) its magical power is made as follows: a piece of the length of a finger of any branch broken off by wind but lodged before it reached the ground, a pinch of the flowers of *Parkia biglobosa* Benth., a bit of the vine of *Piper guineense* Schumach. and some other plants (names unknown) - all is calcined in a pot and triturated with a stick. **Red palm oil** is added and some of the black paste is put into the little horn	a snake doctor brings the prepared cow's tail and he brushes the sick man's face and asks him a question. If he does not answer it means he will die. If he answers the doctor dips his left third finger into a small horn tied to the cow's tail, gets some medicine and rubs it over the patient's heart saying: 'this is my own medicine… I will make you well'. After that the doctor proceeds to make medicine for whatever sickness the patient had to start with	Liberia	Mano	[48]	
E. guineensis*	fruit (red palm oil)	amenorrhea	Three seeds of *Ricinodendron heudelotii* subsp. *africanum* (Müll.Arg.) J.Léonard (cited as *Ricinodendron africanum*) and a quantity of canna blooms *Canna indica* L. (cited as *Canna bidentata*) are beaten together in a mortar, and put into a big spoon. Then a little salt and **red palm oil** (freshly prepared, not refined by heating) is added. Three pebbles are put in the fire and allowed to get hot, then one of these "rocks" is put into the spoon and stirred until it has cooled, and then discarded. The same process is repeated with two other rocks	Woman starts up the ladder-stick towards the loft and stands with both feet on the first notch and she dips her fingers into the spoon and lick off the medicine. She will have her menstrual function restored in two or three days	Liberia	Mano	[48]	
E. guineensis*	fruit (red palm oil)	gonorrheal orchitis	A doctor takes a piece of the bark of *Erythrina latissima* E.Mey with two of the conical thorns still adherent. He scrapes off the inner bark and chews it up with a few grains of *Aframomum angustifolium* K.Schum. (cited as *Amomum melagueta*)	First a doctor tells the patient to 'wash the thing'. Holding the scrotum in both hands he blows his medicine from his mouth onto it, then he breaks off the two thorns from the bark, calcines them and mixes the powder with **red palm oil**, and rubs it on the scrotum. In two or three days the swelling will go down	Liberia	Mano	[48]	
E. guineensis*	fruit (palm oil)	gonorrhea	Bark fibers of *Waltheria americana* L. are twisted into a cord to be worn around the waist. The cord and loin cloth are smeared with an ointment made of the flower stalks of *Cyathula prostrata* Blume, fried black, and ground up with **palm oil**	worn around the waist	Liberia	Mano	[48]	
E. guineensis*	fruit (palm oil)	tinea cruris	Ointment made of some plant (name unclear) and charred big black ants- ground with **palm oil**	ointment	Liberia	Mano	[48]	
E. guineensis*	fruit (palm oil)	chronic ulcers	Leaves of the variety of *Combretum aculeatum* Vent. growing on dry ground are fried in **palm oil** with a finger ring in the pot	the mass is rubbed on the legs and the ring is worn	Liberia	Mano	[48]	
E. guineensis*	fruit (red palm oil)	snake bites	a piece of every sort of thorny shrub or scratchy vine is collected and all calcined in a pot, beaten to a black powder in a mortar and mixed with **red palm oil**. The medicine is put into a horn or into a big acatma snail shell. Only the horn of the black antelope (*Cephalopus niger*) is a taboo for this purpose. Leaves of *Mareya spicata* Baill. are beaten up with clay and a little put in the horn before it is filled with a calcined mixture	a horn decorated with bracelets is carried by the snake man. The medicine is smeared on the legs if going onto the forest at night without the light. If the snake bites the snake man will not be hurt. The medicine is said to kill the snake if rubbed on its head. This medicine is also used as an emergency treatment for any snakebite. A little eaten and rubbed on the wound is thought to be efficient first-aid treatment	Liberia	Mano/Ba Kona	[48]	
E. guineensis*	fruit (red palm oil)	control of snakes	leaves of *Mareya spicata* Baill. are calcined and mixed with **red palm oil**. The black ointment is put into a horn	when a snake is seen on a tree, some of the black ointment is taken and rubbed around the tree trunk saying 'gbaka'. The snake is supposed to fall down out of the tree, and be easily killed. If there is no stick handy to kill the snake with, a person should rub the ointment on both hands, grab the snake by tail and beat it against the ground	Liberia	Mano/Ba Kona	[48]	the author mentions that this procedure is probably all magical except the act of beating the snake on the ground
E. guineensis*	fruit (red palm oil)	protection for women	calcined twig of *Protomegabaria stapfiana* Hutch. (mentioned as *Protomegabaria staphiana*) mixed with **red palm oil** and salt and put into a horn	any woman member of the snake society has a horn of this medicine tied to her waist to keep her from getting sick as a result of her contact with the snake people when she attends a meeting to sing and dance. She may lick the medicine from the end of the finger if she feels dizzy or afraid	Liberia	Mano/Ba Kona	[48]	the author mentions that these practices are highly magical
E. guineensis*	fruit (palm oil)	protective medicine and fetishes	when preparing the Poro session the ritual of feeding the fetish had to be made: with cooked rice, the gizzard cut into bits and some **palm oil**, saying: “(…) Let all people come here so we can be prosperous”	see preparation	Liberia	Mano/Ba Kona	[48]	
E. guineensis*	fruit (palm oil)	malaria		shea butter (*Vitellaria paradoxa* C.F.Gaertn.) is used to make a ring around the neck. Underneath another ring with **palm oil** is made. If a patient is a female, a doctor puts left hand on her head; and right hand if it is a man. Then the following incantation is recited: “The mosquito with six children is the name given to the blacksmith who makes headache (repeat thrice). Two of the children went to a white tree, two went to a Kogbe tree, the last two were sent by Orunmila [the deity] to go and beat the kiriji drum on the heads of human beings. But Orunmila ordered that this drum should not be beaten on the head of those patients that make this mark of shea butter and oil around their neck. Because of this, [name of the patient] whose neck has been marked around with shea butter and oil should be quickly spared”	Nigeria	Yoruba	[47]	the author mentions this treatment is for high temperature and severe headache, but makes a note that this is probably equivalent to Western malariology
E. guineensis*	fruit (palm oil)	smallpox	leaves of Kalanchoe sp., leaves of Peperomia pellucida Kunth. and powdered snail shells are mixed into an oily base consisting of palm oil and shea butter. Preparation of the ointment is accompanied by the long incantation. A particular Ifa sign (from Ifa-oracle divination) should be made upon the surface of the calabash containing the ingredients	the resulting ointment is efficacious in reducing pock marks or scarring	Nigeria	Yoruba	[47]	
E. guineensis*	fruit (palm oil)	offerings	**palm oil** is offered to a variety of *vodun* spirits. For the annual yam celebration, Legba – the guardian spirit, is offered yams, palm oil, chicken blood, and other offerings. Throughout coastal Benin palm oil is also used in *vo*, which are sacrifices or offerings used in daily problem solving. An example of *vo* is a calabash containing kola nuts, palm oil, and other items indicated by the diviner. It is placed in the center of a paved road, and by end of the day it is run over by cars, so the problems are destroyed		Benin		[37]	
E. guineensis*	fruit (palm oil)	offerings	near almost every door there used to stand the Legba-pot, filled every morning and evening with cooked maize and **palm oil**. And upon the *vodun* called the “Vulture’s Dish” the passers-by used to deposit a little food or palm oil, to bring luck or ward off danger		Benin		[50]	
E.guineensis* + Unidentified palm	fruit (red palm oil) + sap (palm wine)	black magic - poison	a bark of a tree from Rutaceae family is mixed with young branches of Mimosa sp. and Byrsocarpus coccineus Schumach., thoroughly roasted in a pot, beaten to powder and mixed with red palm oil and crocodile gall. Kept in a horn of the black antelope. A little of a poison is put under the thumb-nail and placed in the palm wine	see preparation	Liberia	Mano	[48]	
*Dypsis canaliculata*	whole plant (palm tree)		**palm** tree planted at sacred places		Madagascar	Betsimisaraka	[13]	
*Dypsis fibrosa*	leaf	festivities		**leaves** used to decorate houses at clerical festivities	Madagascar	Betsimisaraka	[14]	
*Dypsis pinnatifrons*	leaf	festivities		**leaves** used in decoration of churches, and pinnae to manufacture crosses for churches	Madagascar	Betsimisaraka	[13]	
*Hyphaene coriacea*	leaf	circumcision ceremony		l**eaves** are tied to legs of boys and heads of women during circumcision ceremony	Kenya	Camus	[57]	
*Hyphaene coriacea*	leaf	ritual		**leaves** used to prepare bridal hats	Namibia	Ovambo	[58]	
*Phoenix reclinata*	leaf	ceremonial and religious purposes			Uganda		[59]	
*Raphia farinifera*	leaf	festivities		**leaves** are utilized for making crosses, and they are burned as incent at church	Madagascar	Betsimisaraka	[13]	
*Raphia hookeri*	seed	ritual baby care	the **seeds** of *Raphia hookeri* are used to treat the baby’s fontanel that “beats”. The seeds are roasted over the fire till they are black as coal, ground to powder, mixed with some unknown ingredient (perhaps oil) and the mixture is smeared on the fontanel		Ghana and Benin		[54]	
*Raphia vinifera*	leaf	against witchcraft, or any member who recently had sexual intercourse/preventive	a curtain made of **bud leaves** of *Raphia vinifera*	a curtain is a barrier set up across the road leading to the secret place of a meeting. It is effective against any outsider that may bring witchcraft medicine, poison, or any member who recently had sexual intercourse	Liberia	Mano	[48]	
*R. vinifera*	leaf	to ward off the evil	fresh **bud leaves** are suspended as a curtain in the villages’ entrances to ward off the evil		Cameroon		[van Andel, unpublished]	
Unidentified palm	seed (palm nut)	oracles	palm nuts used in *Afa* divination in Benin. The 16 palm nuts were cleared, marked with certain *Afa* motif, and thrown from right hand to the left to reveal the destined combination		Benin		[50]	
Unidentified palm	seed (palm nut)	oracles	palm nuts used in secret *Fa* divination in order to decrypt and read the signature of god		Benin		[38]	
Unidentified palm	leaf (palm mat made of leaf fiber)	broken limbs/splints	**mats of split palm** are tied around with bark string. The legs of chicken are broken to cure the patient and the chicken together	the patient is segregated from the village in a grass hut. Medicine leaves are applied to the skin under the mats. The legs of chicken are broken to treat the chicken and the patient together. When the chicken starts to walk again so will the patient	Zambia	Lunda	[9]	
Unidentified palm	seed (palm nut)	craw-craw	leaves of *Morinda morindoides* (Baker)Milne-Redh. (cited as *Morinda confusa*) are mashed and made into a leaf packet with two **palm nuts**; the packet is roasted in the fire	the pulp is rubbed on the skin	Liberia	Mano	[48]	the author adds: “the nuts are obviously magical”
Unidentified palm	seed (palm nut)	pterygium	a **palm nut** is carefully cracked and the kernel removed entire; a hole is bored through the kernel; the operator chews up a leaf of *Microdesmis puberula* Hook.f., and holding the palm kernel bead against the white spot on the eye, blows the leaf emulsion into it	the leaf emulsion is blown into the eye. If the lesion is recent it will go away at once	Liberia	Mano	[48]	
Unidentified palm	seed (palm kernel)	impotence	young leaves of *Microdesmis puberula* Hook.f. are chewed with **palm kernels**, while the mass is rubbed on the back of the loins	see preparation	Liberia	Mano	[48]	
Unidentified palm	sap (palm wine)	smallpox	**palm wine** is an important drink for patient, and offering to the Shopanna god	palm wine should be drunk and sprinkled throughout the house to appease Shopanna, but the patient should also be rubbed with it. Relatives should not sleep near an infected person, nor visit anyone outside. Roasted groundnuts should not be eaten during an epidemic, as this would offend Shopanna. No drumming should be performed	Nigeria	Yoruba	[47]	
Unidentified palm	sap (pam wine)	against witchcraft	a small switch of Ixora sp. and a bit of Vernonia conferta Benth are calcined and the powder is put in a small horn; palm wine is added	a snake man rubs his finger in medicine and licks it saying: "if anyone wants to make *wi* for me, let him come to me straight" - meaning that if anyone wants to bewitch him will come foolishly like an intoxicated man and tell him what he has come for	Liberia	Mano/Ba Kona	[48]	
Unidentified palm	sap (pam wine)	black magic - poison	a bark of a tree from Rutaceae family is beaten and partially dried, castings of earth worms are added and all heated thoroughly. While beating the mixture the name of the victim is called and the poison is told to kill the victim in two days. A thumb of the poison is placed in a gourd of **palm wine**	the victim is invited a gourd of palm wine with a thumb of the poison always using the left hand	Liberia	Mano	[48]	
Unidentified palm	sap (palm wine)	sacrifice to various spirits to buy protection	a sacrifice (offering) made of food, cotton, parts of a sacrificial animal and **palm wine**	accompanied by a prayer: “We come to you. We want you to come and eat with us. Here is your part. This cotton is our clothing and our money. This is part of our meat. Here is some palm wine for you. We want you to help us. Bring us good luck, let us have no sickness, let us have plenty of money, let us have good crops and plenty of children. (…) Come and be a god to us and do not let any evil befall us”	Liberia	Mano	[48]	
Unidentified palm	whole plant (palm tree)	charm, selfprotection	a self-protecting charm which involves putting one's life into a hiding place; and some people are doctored to hide in a **palm** tree. When such a one dies, the palm falls; and should the palm fall first (a very unlikely event), the man would die	see preparation	Zambia	Ba-Ila	[45]	
Unidentified palm	fruit	prayer before administratin g the drug	a doctor sits before the patient and holds in one hand the small calabash containing the medicine, and in the other takes a rattle made of round **palm fruit** on a handle, and as he rattles it he prays as follows: “I am humble! It is thou who created this medicine and all things. May this person live. Drive away witchcraft. Let this medicine make him strong. May he see life!”	see preparation	Zambia	Ba-Ila	[45]	
Unidentified palm	leaf (a string made of palm leaf)	preventive against the malign influence of pregnant women	a string made of **palm leaf** is suspended on poles in front of the hut to give warning, especially to the pregnant women. This is to “fend off by means of string”. If a pregnant woman enters a hut where there is a baby its skull would part asunder	see preparation	Zambia	Ba-Ila	[55]	
Unidentified palm	whole plant (palm tree)	a sacred spot associated with the demigod	a bare place about an acre in extent, with a solitary palm-tree growing upon it. It is reckoned as “chikomo”: a word applied to places, rites, and customs traditionally associated with demigods. It is there that the communal gatherings take place before and after war: e.g. where the warriors are doctored	see preparation	Zambia	Ba-Ila	[55]	
Unidentified palm	leaf	protective amulet -against the perspiration of those who have sexual relations	convalescents after a disease are provided with *fowa-* a rattle which consists of a root (unknown plant) contained in a kind of round box made of **palm leaf**	tied around the ankle	South Africa	Thonga	[10]	
Unidentified palm	leaf	possession by spirits -madness		a large **palm leaf** from *Milala* palm is waved in front of a patient – sufficient to “scatter the spirits” which cause madness	South Africa	Thonga	[10]	
Unidentified palm	sap (palm wine)	preventive/offering/sacrifice	palm wine in a big pot called gandjelo (which also means altar) is an offering for ancestor-gods. This is necessary to obtain the favor and help of the ancestor-gods; or to reduce their anger and, therefore, the risk of disease or other calamity brought by displeased ancestor-gods	see preparation	South Africa	Thonga	[10]	
Unidentified palm	leaf	punishment of thieves	a person which is guilty of having stolen the missing property may be punished by confrontation with several **palm leaves**, which, by a kind of supernatural judgment, turn into snakes	see preparation	South Africa	Thonga	[10]	
Unidetified palm	leaf	“when it bites inside” - colic	the remedy is prepared from the roots of unknown plants, cut into equal lengths and tied together with a band of **palm leaf** (a bunch is called *shitsimbo*). The bunch is then boiled to bring out the active principles of the drug	the decoction is taken by a patient just as it is; sometimes it is mixed with maize. The bunch retains medicinal properties for a long time and it may be used during a whole week	South Africa	Thonga	[10]	
Unidetified palm	leaf	festivities	palm skirts used by dancers		Liberia	Poro	[48]	
Unidetified palm	leaf	festivities	palm skirt used by women		Zambia	Mwila	[45]	
Unidetified palm	leaf	vodun	a vodun *Vo-sisa* used to be placed opposite to the house gates to defend the inhabitants from harm. It usually consisted of a pole, with an empty old calabash for a head, and a body composed of grass thatch, palm **leaves**, fowl’s feathers, and snails’ shells		Benin		[50]	
Unidetified palm	leaf	sacrifice	palm **fronds** are used in *kudio*, which are sacrifices used to heal a dying person by exchanging the life of an animal for that of the person		Benin		[37]	
Unidetified palm	leaf	offerings	offerings are made to various *vodun* spirits over a fresh bed of *azan* - ritual palm **fronds** which mark the sacred spot		Benin		[37]	
Unidetified palm	leaf	protective	the *azan* (ritual palm **fronds**) was worn around the throat, to protect from witchcraft or from being killed during war		Benin		[50]	
Unidetified palm	leaf	punishment	palm **fronds** are carried by people involved in punishing social deviants, and those suspected of witchcraft		Benin		[38]	
Unidetified palm	leaf	punishment	during the ‘witch parades ‘organized to punish and march the accused Beninese women to prison, the witches are bedecked in wreaths of palm **fronds**		Benin		[39]	
Unidetified palm	sap (palm wine)	offering	sodabi which is a locally distilled palm wine is used in offerings made to vodun spirit called Tchamba – an old spirit based on domestic African enslavement		Benin		[63]	

* scientific name not mentioned/specified by the author – assumption made based on the palm part used; as follows:

●coco-nut a ➜ *Cocos nucifera*.

●palm oil/red palm oil x➜ *Elaeis guineensis*.

The extracted information was standardized to generally accepted terminology for palm morphology following Dransfield et al. [40]. When oil was given as a palm part used and the author did not mention the scientific name of the palm it was assumed that palm oil was extracted from the African oil palm *Elaeis guineensis,* since processing of the fruits for edible oil has been traditionally practiced in Africa for thousands of years [41]. Medicinal uses referring to “coconut” were assigned to *Cocos nucifera*. Some palms remained unidentified, such as those used for “palm wine”, which can be produced from various species including *Elaeis guineensis*, *Hyphaene* spp., *Raphia* spp., as well as *Phoenix reclinata*[41]. Country names referred to in the older literature which are no longer in use, were updated to the current names, i.e., Northern Rhodesia to Zambia, Dahomey to Benin. Species and author names coincide with the World Checklist of Palms [42] and The International Plant Names Index (IPNI) [43] for other plant species. The authors of scientific names of palms are included in Table 2, and authors of other plant species in the (Table 1).

**Table 2:** Ritual uses of palms in sub-Saharan Africa

**Palm species**	**Palm part(s) used**	**Purpose**
*Borassus aethiopum* Mart. African fan palm	seed, root	- treatment of diseases and disorders
		- ceremonies
*Cocos nucifera* L. Coconut	fruit, entire palm tree	- treatment of diseases and disorders
		- sacred places
		- offerings
*Dypsis canaliculata* Jum. (Beentje) & J.Dransf.	entire palm tree	- sacred places
*Dypsis fibrosa* (C.H.Wright) Beentje & J.Dransf.	leaf	- ceremonies
*Dypsis pinnatifrons* Mart.	leaf	- ceremonies
*Elaeis guinnensis* Jacq*.* African oil palm	fruit, seed, infructescence/inflorescence, leaf, root	- treatment of diseases and disorders
		- offerings
		- protection
		- oracles
		- taboo
*Elaeis guineensis* var. *idolatrica* A.Chev.	entire palm tree	- sacred places
*Hyphaene coriacea* Gaertn. Doum palm	leaf	- treatment of diseases and disorders
		- ceremonies
*Hyphaene petersiana* Klotzsch ex Mart.	leaf	- ceremonies
*Phoenix reclinata* Jacq. Senegal date palm	leaf	- ceremonies
*Raphia farinifera* (Gaertn.) Hyl.	leaf	- ceremonies
*Raphia hookeri* G.Mann & H.Wendl. Raphia-palm	seed	- treatment of diseases and disorders
*Raphia vinifera* P.Beauv. West African piassava palm, Bamboo-palm	leaf	- protection
Unidentified palm species	leaf	- treatment of diseases and disorders
		- offerings
		- protection
		- oracles
		- ceremonies

The purposes are generalized into categories.

## Results

**Figure 1:**
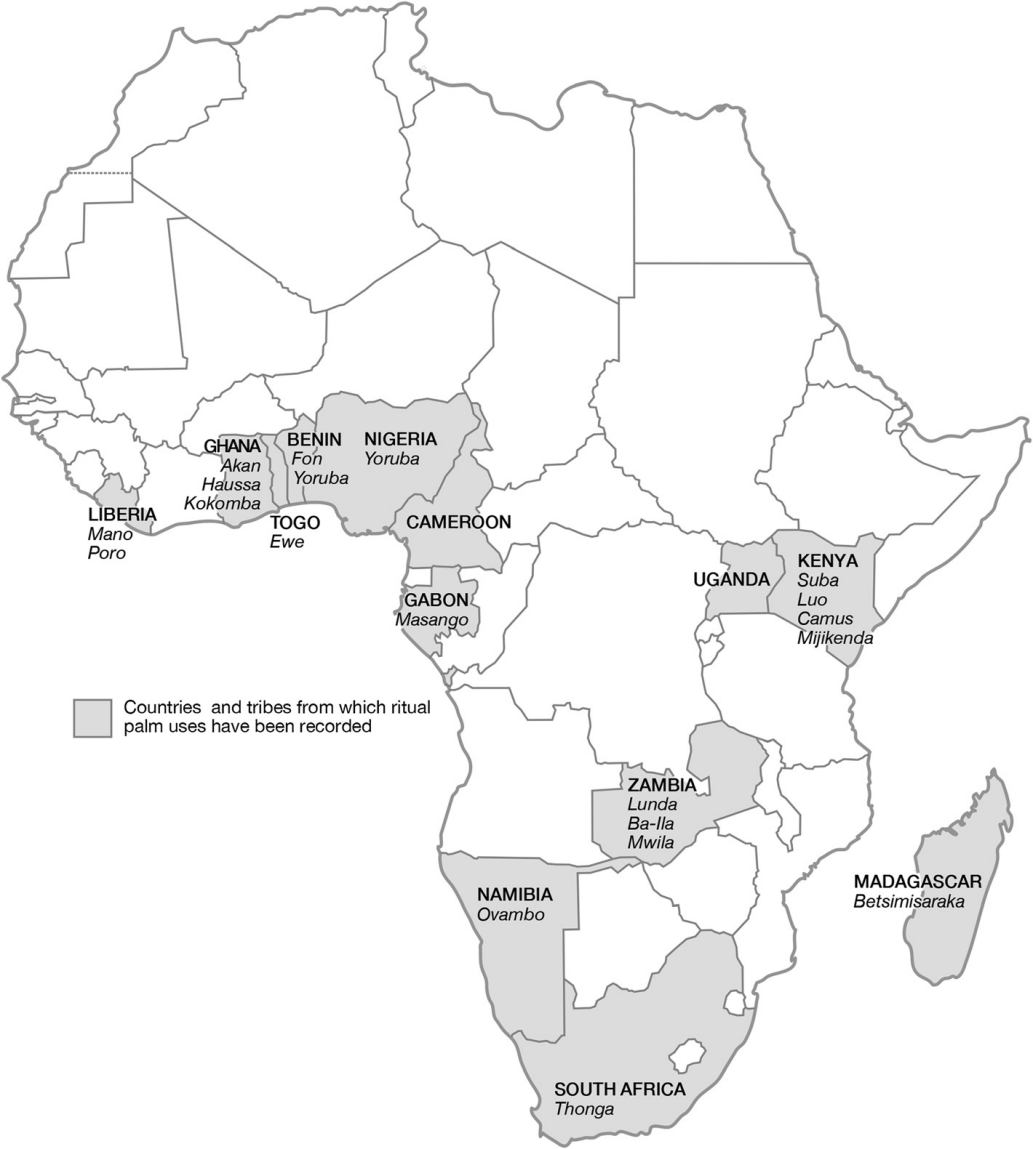
**Localities in sub-Saharan Africa where ritual uses of palms have been reported.** In total 81 ritual uses of at least 12 palm species have been reported in 13 countries and 19 ethnic groups.

We found references to ritual uses of at least 12 palm species in sub-Saharan Africa, and they were used for 81 different purposes (for a complete list see the (Table 1). Ritual uses of palms were encountered for 13 different countries and 19 different ethnic groups in the region (Figure 1). The results were organized following the part of palm used.

The palm fruit is made up of three carpels that fuse to form a drupe with one or a few seeds, covered by a thin seed coat. The seed consists mostly of a large oily endosperm which when immature is watery and gelatinous before it turns hard in the mature seed. The seed has a small embryo near one of the germination pores which are thin areas of the bony endocarp. Palm seeds are often called palm kernels or palm nuts [44]. The seed(s) is (are) surrounded by a three layered pericarp consisting of an outer leathery exocarp, a middle fleshy and oily or fibrous mesocarp, and an inner endocarp which may be thick and bony or thin and papery. Palm oil may be extracted from the fleshy mesocarp or from the endosperm of the seed in which case it is called kernel oil. Red palm oil, extracted from *Elaeis guineensis* fruits, gets its characteristic color from carotenes in the mesocarp, although it can be bleached to produce colorless oil [41,44].

Palm leaves usually are assembled in a rosette at the end of the stem. Each leaf consists of a sheath, a petiole, and a lamina which in turn is made up of a midvein (rachis) and several leaflets (pinnae). In some palms (*Cocos*, *Elaeis, Phoenix, Raphia*), the midvein is elongate and has several leaflets that form a pinnate leaf. In other palms (*Borassus, Hyphaene*), the rachis is reduced and all leaflets radiate from a single point to form a palmate lamina [44]. The uses mentioned below either refer to whole palm leaves or to leaflets, which are dried and used for weaving. In some cases fibers are extracted from the leaflets and used for weaving.

Palms produce a sugary sap for their growth, which is often tapped by humans and used to prepare a fermented beverage called palm wine. Palm sap is extracted from different species, including *Cocos nucifera, Borassus aethiopum,* and *Elaeis guineensis.* Tapping is done by cutting the inflorescence and collecting the sap from the injured peduncle or inserting a tube into the palms growing point in the heart of the crown and placing a container at the end of the tube to collect the sap [41].

Roots of palms are adventitious: they originate from the lower part of the stem and are not part of a tap-root system. Therefore, roots can be collected from the palm without the need to dig them out of the ground, and subsequently employed for a variety of purposes [44].

### Fruit (palm oil)

In Zambia, palm fruits were used in prayers before administrating a drug to ensure the effectiveness of medicine and successful recovery of a patient. The Ba-Ila healer used a rattle made of round palm fruits on a handle during ritual therapies [45]. To the Lunda in Zambia, the red color of the mesocarp oil from *Elaeis guineensis* symbolized power, but it also was interpreted a sign of murder and witchcraft [9].

On Mfangano Island in Kenya, the Suba and Luo still use the fruits of *Cocos nucifera* to alleviate skin rash associated with HIV/AIDS. The disease – locally known as ‘chira’, and its etiology is related to the transgression of principles governing sexuality or seniority. These include adultery committed during a wife’s pregnancy, having sexual intercourse during the harvest, or failure to observe the proper separation of sexuality between generations [46].

Disorders of the reproductive system that fail to respond to rational therapy are often explained by witchcraft or broken taboos [7,47]. In Nigeria, to prevent miscarriage Yoruba people used to roast a tortoise with coconut water and half a bottle of palm oil from *Elaeis guineensis*, after which the mixture was ground to powder [47]. The powder was consumed in a corn flour pudding, taken every morning and evening during one menstrual period, followed by sexual intercourse five days after finishing menstruating. The Yoruba saw the tortoise as a symbol for a prostitute, which might signify that when a woman suffered miscarriage it could be due to committed adultery or a broken taboo [47]. Sex taboos in particular were often used as an explanation for the occurrence of a disease [45,48]. For the treatment of malaria, the Yoruba used start the ritual with two rings painted on the neck, one with shea butter (*Vitellaria paradoxa*), and another with *Elaies guineensis* oil [47].

In Liberia, the Mano used red palm oil in treatments of mysterious diseases. To awake a patient in coma, red palm oil was mixed with a burned knot of the parasite *Loranthus micranthus* and rubbed on the patient’s cheeks toward the mouth in order to make him talk (all information in this paragraph derived from [48]). Another magical medicine was prepared from any branch broken off by wind but lodged before it reached the ground, mixed with some burned plants and *Elaeis guineensis* oil. The paste was put into a little horn tied to a cut off cow’s tail. The traditional healer asked the sick man a question while brushing his face with the tail. If there was no answer, it meant that the patient would die. If he answered, the healer took some medicine with his left third finger, and rubbed it over the patient’s heart while saying specific prayers. The broken but not fallen branch perhaps symbolized hope for the patient to wake up. The palm oil was used to blend the various added ingredients. Similar symbols appeared when healing fractures in Liberia. A few branches from various shrubs and trees were gathered, together with any broken twig which was healed but growing in a twisted position. The charred wood was mixed with red palm oil, and the ointment was then applied to the fracture. The twisted branch probably symbolized the twisted limb. Another example of sympathetic magic among the Mano was a cure for acute hepatitis, in which shelf fungi shaped like a liver were mixed with *Elaeis guineensis* oil and rubbed over the liver. In a cure for palpitation, the Mano mixed red palm oil with an inflorescence of *Costus* sp. and a handful of buds from *Harungana madagascariensis*. Part of the mixture was put in an iron spoon with four pebbles that had been heated in the fire. There would be three pebbles used if the patient was a female. Notably, the stone represented longevity and strength, and the red color of palm oil as well as the red sap from *H. madagascariensis* probably symbolized the color of the treated heart. Gender specific symbolic medicines were also used in Liberia for rheumatism due to yaws. Enchanted elements were the leaves and bark of living plants and red palm oil, which represented the active male elements. The charred plants and “burned” oil represented the soothed, magical, more preventive female elements. Male and female medicines were mixed in order to form even more powerful Mano medicine, and possibly to achieve an ideal balance between active and controlled features of persons and situations. Women in Liberia used to protect themselves from getting sick during the meetings of the Ba Kona snake society using a medicine horn made of charred twig of *Protomegabaria stapfiana* and red palm oil. The medicine was tied to the woman’s waist and licked from the finger whenever she felt dizzy or afraid. Although in many ritual medicines palm oils served merely as a rubbing agent, the use of the oil could also be a taboo. In Liberian Sukba Society it was forbidden to put red *E. guineensis* oil into a horn of medicine, which was a fetish prepared to protect from witchcraft. If the taboo was broken, the fetish could turn against its owner; catch him and kill him instead of the witch [48].

In Gabon, the Masango used the leaves of *Hyptis lanceolata* mixed with palm oil to apply on the body as medico-magic [49]. Palm oil is still offered to a variety of *vodun* spirits in Benin [37]. For the annual yam celebration, the guardian spirit Legba receives yams, palm oil, chicken blood, and other offerings. Throughout coastal Benin, palm oil is also used in *vo*, which are sacrifices or offerings used in daily problem solving. An example of *vo* is a calabash containing kola nuts, palm oil, and other items indicated by the diviner. It is placed in the center of a paved road, and by end of the day it is run over by cars, so the problems are destroyed [37]. In Benin near almost every door there used to stand the Legba-pot, filled every morning and evening with cooked maize and palm oil [50]. For another *vodun* called the “Vulture’s Dish”, passers-by used to deposit a little food or palm oil, to bring luck or ward off danger [50].

### Seed

In Liberia, palm kernel oil was applied with owl’s feathers on wounds resulting from scarification performed during the Poro initiation rites [48]. In Cameroon, fresh seeds of *Elaeis guineensis* were mashed and mixed with other plants to treat mental fatigue. The residues of medicine were put under the pillow, and only if the patient dreamed of a young girl with erected breasts, there was hope for cure [51]. *Elaeis guineensis* seeds were also used as sacred objects in rituals involved in oracles, which helped to discover the cause of disease or other calamities for example the *Afan* oracle of the Ewe in Togo, or the *Ifa* oracle of the Yorubas and *Fa* oracle of the Fon in Benin [51]. In the *Afa* divination in Benin, 16 palm nuts were cleared, marked with certain *Afa* motif, and thrown from right hand to the left to reveal the destined combination [50]. In 1990s palm nuts were still used in Benin in secret *Fa* divination in order to decrypt and read the signature of Fa – a god of oracles [38].

Craw-craw, is the local term for onchocerciasis or river blindness, which causes itchy rashes and nodular swellings on the skin. In Liberia crawcraw was threated with mashed leaves of the brimstone tree *Morinda lucida* made into a leaf packet with two palm seeds. The packet was roasted in the fire and the pulp rubbed on the skin. According to Harley [48], p. 92 “The nuts obviously are magical”. Among the Mano in Liberia, impotence, which was widely believed to be caused by witchcraft, used to be treated with palm seeds chewed with young leaves of *Microdesmis puberula,* and the mass was then rubbed on the genitals [48]. The presence of *M. puberula* in the treatment for impotence might be linked to its wood which is very hard [52].

In Ghana, hollowed out *Borassus aethiopum* seeds are still used by Haussa’s as containers for a charred medicinal mixture called ‘katala’ in Haussa. This mixture is rubbed into skin incisions during scarification practices [11].

The seeds of *Raphia hookeri* were sold in 2010 and 2011 on markets in Ghana and Benin to treat a baby’s fontanel when it “beats”, which is seen as an unhealthy symptom [11,53]. The seeds are roasted over the fire until they are black as coal, ground to powder, mixed with oil and the mixture is smeared on the fontanel. The seeds are also boiled as tea or added to herbal baths to treat babies with beating fontanels [54].

### Inflorescence/infructescence

In Ghana, empty infructescences of *Elaeis guineensis* alone or mixed with ginger (*Zingiber officinale*) are burned and applied as magical medicine in the form of an enema to small children to encourage them to walk at an early age [van Andel, unpublished]. Also in Ghana, the Akan burn inflorescences from *Elaeis guineensis* so the smoke drives away bad spirits [7,11].

### Leaves

In Zambia splints made of palm mats used to be tied around broken limbs with bark strips, and medicine was applied on the skin under the mats [9]. Simultaneously, the legs of a chicken were broken and treated with the same medication. The Lundas believed that when the chicken starts to walk again, so will the patient. Palm leaves were used as protective barriers by the Ba-Ila. A string made of fibers extracted from the palm leaf was suspended on poles in front of the hut to warn pregnant women. If a pregnant woman entered a hut where there was a baby, its skull would part into pieces [45,55].

During secret meetings of the Mano in Liberia, a curtain of young *Raphia vinifera* leaves protected from any outsider who could perform witchcraft, bring poison, or any person who recently had sexual intercourse [48].

In Cameroon, fresh bud leaves of *Raphia vinifera* are still suspended as a curtain in the villages’ entrances to ward off the evil [van Andel, unpublished].

In Benin a vodun *Vo-sisa* used to be placed opposite to the house gates to defend the inhabitants from harm. It usually consisted of a pole, with an empty old calabash for a head, and a body composed of grass thatch, palm leaves, fowl’s feathers, and snails’ shells [50].

Palms were also used as protective amulets. In Benin and Togo, ill people carried twigs of *Elaeis guineensis* around the neck or arm to achieve invulnerability [51].

In South Africa, during the *hondlola* purification ceremony, performed after a cure was accomplished, the Thonga protected themselves from perspiration of those who had sexual relations. A convalescent was provided with *fowa*, which was a kind of round rattle made of palm leaf tied around the ankle. Madness, which the Thonga associated with spirit possession, was treated by waving a large palm leaf from the milala palm (most probably *Hyphaene coriacea*) in front of the patient, which would “scatter the spirits”. Also “when it bites inside” a medicine was prepared from various equally cut roots that were tied together with a band of palm leaf, and boiled to bring out the active principles of the drug [10].

Palm fronds are still used in Benin in *kudio*, which are sacrifices used to heal a dying person by exchanging the life of an animal for that of the person [37]. Also, offerings are made to various *vodun* spirits over a fresh bed of *azan* - ritual palm fronds which mark the sacred spot [37]. The *azan* was also worn around the throat, to protect from witchcraft or from being killed during war [50]. In Benin, palm fronds are also carried by people involved in punishing social deviants, and those suspected of witchcraft [38]. During the recent ‘witch parades’ organized to punish and march the accused Beninese women to prison, the witches were bedecked in wreaths of palm fronds [39]. Perhaps palms bring justice because they are associated with understanding, peace, and harmony, or with indwelling tree spirits themselves [56]. In South Africa a thief was punished by confrontation with palm leaves, which, by a kind of supernatural judgment, turned into snakes [10].

Palm leaves also served in various ceremonies, rituals and religious festivities. In Kenya, the Camus tied the leaves of *Hyphaene coriacea* to boys’ legs and heads of women during the circumcision ceremony [57]. Skirts made from palm leaves were used by the masked Poro dancers, and by the Mwila women on festive occasions [45,48]. Leaves of *Hyphaene petersiana* were used in Namibia to prepare bridal hats among the Ovambo [58]. Nowadays, in Uganda, leaves of *Phoenix reclinata* are used for ceremonial and religious purposes [59]. Betsimisaraka people of Madagascar use leaves of *Dypsis pinnatifrons* in decoration of churches. Leaves of *Raphia farinifera* are used for making crosses, and they are burned as incense at the church [12]. Entire leaves of *Dypsis fibrosa* are used by the Betsimisaraka to decorate houses at clerical festivities [14]. Carrying the palm fronds on Palm Sunday is an important Christian tradition practiced now in many parts of Africa [60,61].

### Sap (palm wine)

In Nigeria, to recover from smallpox, palm wine was drunk and rubbed on the body of the patient. Relatives were advised not to sleep near an infected person, nor visit anyone outside. Roasted groundnuts (*Arachis hypogaea*) were not to be eaten during an epidemic, and no drumming could be performed. These activities would offend the Shopanna god who, according to Yoruba beliefs, was responsible for bringing smallpox epidemics upon mankind. For successful recovery it was also necessary to make an offering to the Shopanna god by sprinkling palm wine all over the house to appease the god [47]. Ancestor spirits appreciate drinks, and palm wine was often used in offerings and fetishes to obtain their favor and help or to reduce their anger and, therefore, the risk of disease or other calamity [10,48]. To engage a powerful being in a relationship of beneficial exchange and prosperity, palm wine was a valued consumable and lubricant of good relations and hospitality. In Kenya, Mijikenda people used to place the coconut shells filled with palm wine on ancestors’ graves as an offering [62]. In Benin, *sodabi* which is a locally distilled palm wine is still used in offerings made to vodun spirit called Tchamba – an old spirit based on domestic African enslavement [63].

The Mano of Liberia carried amulets for protection against witchcraft, made from small horns with enclosed palm wine and charred and powdered twig of *Ixora* sp. and *Vernonia conferta*. By licking the medicine from the finger, a person was ensured that if anyone wanted to bewitch him, he would foolishly turn himself out, and would subsequently be called to account [48]. Although most medicines were directed toward the cure and prevention of disease, some could also embrace poisons [5,9,10,64]. In Liberia, a poisonous mixture was prepared while calling the name of the victim, put under the thumbnail and then placed in a gourd of palm wine. The victim was offered the lethal drink, always using the left hand [48].

### Root

Ritual uses of roots are few. Roots of *Elaeis guineensis* in mixture with the resin from *Daniellia oliveri* and *Commiphora africana* were reported to keep away bad spirits in West Africa [51].

In Ghana, a decoction from *Borassus aethiopum* roots is still used by Kokomba traditional healers in the treatment of any disease caused by a curse [Gruca, unpublished]. In Togo, macerated roots from *B. aethiopium* are used in herbal baths, and powdered or decocted roots from *E. guineensis* are used orally to treat epilepsy [65]. It is believed that epilepsy occurs mostly during the full moon. Mysterious and spontaneous diseases such as epileptic seizures are often associated with supernatural forces [66].

### Entire palm tree

Palm trees played a protective role in Zambia. According to Ba-Ila beliefs, a person could be guarded from harm during the entire life by hiding one’s life in a palm tree. This protective ritual ensured that only if the palm tree would fall the person would die, and since this event was considered unlikely, a palm tree was a safe place to hide one’s life [45]. That might be because the palm tree, due to its unchanging beauty of the evergreen foliage, is considered a symbol of everlasting life, permanence and strength [67]. In Zambia, solitary growing palm trees also provided sacred places where rites and customs traditionally associated with half-gods were performed [55].

The French botanist Chevalier [68] published a varietal name of the African oil palm (*Elaeis guineensis* var. *idolatrica*) referring to its divine characters. Current taxonomy treats this name as a synonym of *E. guineensis*[42] but it is still mentioned as the “idolatrica” palm in some literature even if it cannot be recognized taxonomically. In Benin, the “idolatrica” palm has been recently reported as sacred and protected where ever it grows because it is seen as the realization on earth of the god *Fa*. Nobody is allowed to cut it down or to use its fruits for making oil. The ritual use of these palms is reserved for soothsayers called *bokonon*[69].

In Madagascar *Cocos nucifera* and *Dypsis canaliculata* are still planted at sacred places by the Betsimisaraka people [13]. The Wanaka of East Africa believed that the coconut palm has a spirit, and destruction of this tree is equivalent to matricide because the coconut tree gives people nourishment, as a mother nourishes her child [56].

## Discussion

At least 12 palm species were found to be involved in various ritual practices (Table 2). Assuming that palm oil or red palm oil always come from *Elaeis guineensis*, this was the most commonly documented palm species for ritual purposes in Africa (see Table 1). Because several ritual uses of palms listed in the literature could not be unequivocally referred to a particular species, the picture we draw remains somewhat incomplete with regards to the taxonomic basis for ritual palm uses.

All parts of the palms were used in rituals, but the most commonly used part was the leaf, followed by the fruit and oil extracted from the fruit mesocarp, seed (and extracted palm kernel oil), entire palm tree, sap in the form of palm wine, root, inflorescence and infructescence. The ritual uses of all the mentioned parts were found in both older and recent literature, although most of the recent ritual use records were associated with the palm leaf.

In general, the ritual uses of palms play a double role. In some treatments, the palm is the actual sacred object or the central element of ritual practices, for example entire palm trees determine sacred places, palm seeds accompany oracles and palm leaves serve in offerings. In other cases, palms are used in mixtures with other plants or products. Hence, palms are not the primary ingredient, but support the ritual treatments in some way, like in the case of palm oil, used as a medium to blend and make coherent the various ingredients. It is noteworthy that palm wine and palm oil, which are commonly consumed in Africa, often assist the treatment and the contact with the supernatural world.

Our review shows that palms have been, and probably still are pervasive in African medicinal systems. Their use in medicines reflects the spiritual framework of traditional medical practices, and palms themselves are important and often crucial in disease treatment and prevention. Palm-derived medicines work not only upon diseases of the body, but also directly upon people’s psyche and emotions. Palm medicines can act from a distance if so directed by the power of the spoken word. Sometimes taboos must be respected to secure efficacy. Ritual practices merge in all sorts of combinations with palm remedies. There is no assurance that any particular palm used for the treatment of a particular disease has any biologically active component; it may only be its symbolic or spiritual meaning that serves as a powerful ingredient. Some medicinal plants, just like placebo, can be efficacious without biologically active components [70].

The few studies we reviewed that explained the ritual uses of palms in detail were classic anthropological works that embraced studies of the entire native African tribes and cultures e.g. [5,9,10,45,47,48,55]. The best accounts of traditional medical practices came from those who spent years among the local people, not only observing but also sharing their everyday life. Many of the palm uses mentioned in our review came from these sources, but in many cases we do not know whether the cited rituals are still practiced today, as recent studies on African ritual plants are scarce.

One of the obstacles of ethnomedicinal studies is that little attempt is made to show a more comprehensive meaning of illness, which follows the use of alternative diagnoses and therapies in traditional medical practices. Disease episodes are usually presented only briefly and unambiguously disregarding socially important outcomes that may underlie it. Therefore, it is difficult to get an idea of what, from an emic medical point of view, is going on [71].

It is necessary to remember that the belief systems, which have developed over many generations, form the background to African medical treatments. Continuous interactions with the spiritual world are axiomatically absorbed in childhood, and subsequently reinforced in every phase of life. In fact, it is fascinating that ritual uses of palms are not only present in medicinal practices but in many other events practiced since time immemorial until today.

Knowledge of medicinal plants combined with spirituality continues to thrive in Africa today [31]. Some recent ethnobotanical field studies confirm that divination still plays a major role in the traditional knowledge systems, and palms are still used for this purpose just as they were many years ago [38,51,72]. The belief in witchcraft, divination and spiritual healing has come to coexist with Christianity, independence and development [65,73-76]. While in Cameroon palm fronds are carried by Christians on Palm Sunday [60], they are also used to ward off the evil in village entrances [van Andel, unpublished]. Palms are still considered sacred objects, assuring protection from malevolent forces [37,38,63,69].

## Conclusions

Palms are used in various prescriptions which include a ritual ingredient or procedure. It is impossible to understand the meaning and use of palms in African healing without seeing these uses as part of overall cultural systems, in which techniques of healing cannot be limited to bio-physical ailments or ideas of intervention. In local terms, food and medicine is not strictly separated, and palm products operate in many ways that cannot be isolated from the larger ensembles of elements and practices of which they are part. Effort must be made to provide meticulous reports on traditional remedies, as the enduring value of African medicines lies not only in the materials used, but also in the methods and the concepts underlying them.

## Competing interests

The authors declare that they have no competing interests.

## Authors’ contributions

This study is part of MG’s PhD study under supervision of HB. MG collected the data and wrote the first draft of the manuscripts which was subsequently edited and modified in several rounds by HB; TvA has contributed content and form to the later versions of the manuscript. All authors read and approved the final manuscript.
